# Activin A enhances neurofunctional recovery following traumatic spinal cord injury by inhibiting autophagy

**DOI:** 10.4103/NRR.NRR-D-24-01021

**Published:** 2025-03-25

**Authors:** Liqun Yu, Zhaoyang Yin, Ruiqi Huang, Zhibo Liu, Yuchen Liu, Xinxin Zheng, Simin Song, Zhaojie Wang, Xiaolie He, Yuxin Bai, Li Yang, Xu Xu, Bairu Chen, Jian Yin, Yanjing Zhu

**Affiliations:** 1 Key Laboratory of Spine and Spinal Cord Injury Repair and Regeneration of Ministry of Education, School of Medicine, School of Life Science and Technology, Tongji Hospital Affiliated to Tongji University, Tongji University, Shanghai, China; 2 Clinical Center for Brain and Spinal Cord Research, Tongji University, Shanghai, China; 3 Department of Orthopedics, The First People’s Hospital of Lianyungang, The Affiliated Lianyungang Hospital of Xuzhou Medical University, Lianyungang, Jiangsu Province, China; 4 Department of Orthopedics, The Affiliated Jiangning Hospital of Nanjing Medical University, Nanjing, Jiangsu Province, China

**Keywords:** Activin A, autophagy, cell differentiation, motor function recovery, neural regeneration, neural stem cell, neuroprotection, phosphoinositide 3-kinase/protein kinase B pathway, spinal cord injury, transforming growth factor-β superfamily

## Abstract

In the early stages of traumatic spinal cord injury, extensive accumulation of autophagosomes creates a neurotoxic microenvironment, exacerbating neuronal cell death and worsening tissue damage, ultimately hindering neurofunctional recovery. Activin A is a critical growth factor necessary for the development of the embryonic nervous system and for maintaining neuronal function in the adult cerebral cortex. It can inhibit excessive autophagy in ischemic stroke to reduce neuronal damage. However, the specific mechanism through which Activin A functions in the spinal cord remains poorly understood. In this study, we administered different concentrations of Activin A to neural stem cells from the spinal cord and found that Activin A stimulated the proliferation and neuronal differentiation of neural stem cells. Then, we established an *in vitro* oxidative stress model by using hydrogen peroxide to stimulate the neural stem cells-induced neurons. We found that Activin A could reduce apoptosis caused by oxidative stress. Subsequently, we treated a mouse model of spinal cord contusion with intrathecal injection of Activin A. Behavioral and electrophysiological results showed that Activin A promoted recovery of motor function and reconstruction of neural circuits in the model mice. Finally, RNA sequencing indicated that Activin A inhibited autophagy by activating the PI3K/AKT/mTOR pathway and upregulating the expression of synaptogenesis-related factor Sema3A in the spinal cord. These results suggest that Activin A may mediate the excessive autophagic response after spinal cord injury, promote the reconstruction of damaged neural circuits, and restore neurological function in the injured spinal cord.

## Graphical abstract

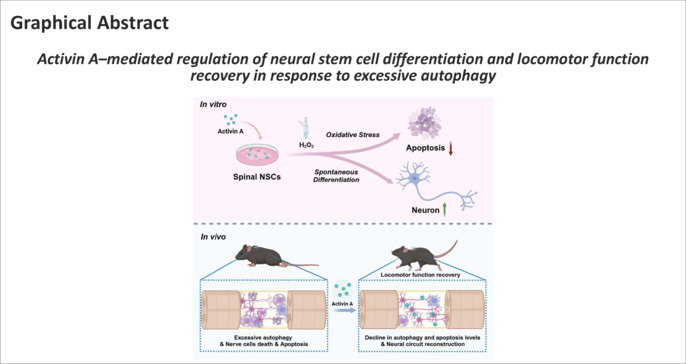

## Introduction

Traumatic spinal cord injury (SCI) is a severe neurological disease characterized by neuron and nerve fiber damage, resulting in impaired neuronal activity and motor function (Nowrouzi et al., 2017; Khadour et al., 2024). Global incidence of SCI ranges from 250,000 to 1 million cases annually, with approximately 22.6 million new cases requiring neurosurgical evaluation and 13.8 million requiring surgical intervention (Zhou et al., 2024). SCI significantly impacts the physical and mental health of patients, in addition to imposing a substantial financial burden on their families and society (Nowrouzi et al., 2017). However, because of the complex microenvironment of lesions, traditional clinical treatments including medication, surgery, and rehabilitation training, are significantly limited in how well they can help restore injured nerve tissue and recover locomotor function (Ahuja et al., 2017).

Cytokine therapy employs biologically active molecules to interfere with disease processes (Cong et al., 2025). It has emerged as a promising therapeutic strategy for treating diseases of the central nervous system (CNS) and in recent years has provided new insights into SCI repair (Azodi and Jacobson, 2016; Deckers et al., 2023). Studies indicate that certain inflammatory cytokines, such as interleukin-10, interleukin-4, and transforming growth factor (TGF)-β1, may play a role in the repair of spinal cord injuries by regulating cellular processes that help heal and restore tissue (Hellenbrand et al., 2021; Li et al., 2023). The TGF-β superfamily is a multifunctional group of cytokines that various tissues can produce. TGF-β has been shown to regulate biological processes such as the cell cycle, cell proliferation, cell differentiation, cell migration, and apoptosis in ways that support treatment of disease (Meyers and Kessler, 2017). Both preclinical and clinical studies have demonstrated that Activin A (AA), a member of the TGF-β superfamily, is involved in the regulation of CNS development and plays a neuroprotective role in the treatment of CNS disorders by regulating inflammation, trauma repair, fibrosis, and cell apoptosis (Ganea et al., 2012; Stayte et al., 2017; Su et al., 2018). As a neurotrophic factor, AA significantly increased the number of neurons without affecting astrocyte differentiation or triggering apoptotic death in neural progenitor cells under conditions that promote differentiation (Rodríguez-Martínez et al., 2012). By regulating both sonic hedgehog and retinoic acid signaling, AA was shown to promote the differentiation of telencephalic neural precursors into postmitotic neurons and inhibit pathways that would otherwise act to maintain the progenitor state (Cambray et al., 2012). Long-term treatment of neurodegenerative diseases, such as Huntington’s disease, with AA protected damaged striatal interneurons and striatal projection neurons (Hughes et al., 1999). Previous studies have also shown that activation of endogenous AA secretion reduces neuroinflammation and stimulates oligodendrocyte progenitor cell differentiation, which in turn alleviates secondary damage after the onset of SCI (Miron et al., 2013; De Berdt et al., 2018). Evidence from treating preterm brain injury indicated that AA influences the differentiation and survival of oligodendrocyte progenitor cells by targeting the Noggin/bone morphogenetic protein 4/inhibitor of DNA binding 2 signaling pathway, which is crucial for regulating cellular processes that contribute to brain recovery after injury (Su et al., 2023). A recent study concluded that AA activates the activin A receptor type-1B signaling pathway, leading to the proliferation and differentiation of oligodendrocytes, an increase of white matter remyelination, and restoration of neurotransmission, ultimately improving neurological function after stroke (Zheng et al., 2021). Meanwhile, a mouse model of cerebral ischemia/reperfusion injury showed that AA significantly enhanced the expression of nuclear factor erythroid-2-related factor 2, which proved pivotal in combating neuronal ferroptosis (Wang et al., 2023). However, despite the numerous reports showing the therapeutic benefits of AA in treating CNS diseases, the underlying molecular mechanisms through which AA functions in treating SCI remain unclear.

Autophagy is a complex biological process that maintains intracellular homeostasis and enhances cell survival by eliminating dysfunctional organelles and abnormal proteins (Shacka et al., 2008; Eisenberg-Lerner et al., 2009). Nevertheless, excessive autophagy is not cell-protective. Dysregulated autophagy can lead to abnormal cell proliferation and development, ultimately leading to programmed cell death (Shen et al., 2012). Analyzing alterations in autophagic flux across various CNS injury models revealed that multiple factors, such as the type and severity of the injury, affect autophagosome formation (Zhang et al., 2020). Two studies indicate that the balance between autophagosome generation and degradation is disrupted after SCI, leading to impaired autophagic flux, which is toxic for neurons (Wu et al., 2021; Rong et al., 2022). In a model of ischemic stroke, AA was shown to activate the phosphoinositide-3-kinase (PI3K)-protein kinase B (PKB/AKT) pathway by binding to the ACVR1C receptor. This inhibited excessive autophagy mediated by the cyclic GMP–AMP synthase–stimulator of interferon genes (cGAS–STING) pathway, thereby reducing neuronal damage and promoting cell survival (Liu et al., 2023).

In the current study, we investigated the role of AA in SCI therapy by administering AA to a mouse model of spinal cord contusion via intrathecal injection. Specifically, we wanted to know whether exogenous AA would affect cellular oxidative stress–mediated apoptosis and excessive autophagy. We further aimed to clarify the role of AA in promoting motor function recovery and neural circuit remodeling in SCI mice and to identify the molecular mechanisms of AA’s action in this regard.

## Methods

### Cell culture

All animal experimental procedures were approved by and performed following the standards set by the Animal Welfare Committees of Tongji Hospital Affiliated to Tongji University in Shanghai (approval No. 2022-DW-SB-46) on February 24, 2024. All animals were provided by Shanghai JieSiJie Laboratory Animal Company (license No. SYXK (Hu) 2022-0017).

Spinal-derived neural stem cells (sNSCs) were isolated from three embryonic Sprague‒Dawley rats at embryonic day 14 (E14) (Zhang et al., 2023b). The pregnant rats were anesthetized with 2% to 3% isoflurane (RWD, Shenzhen, China, Cat# R510-22-10) and promptly transferred to ice. The fetuses were extracted via abdominal dissection. The entire sNSC extraction process was conducted on ice. NSCs were cultured in Dulbecco’s modified Eagle medium/nutrient mixture F-12 medium (Gibco, Grand Island, NY, USA, Cat# 11320033) with 1% B27 (Gibco, Cat# 17504-044), 2% N2 (Gibco, Cat# 17502-048), 1% penicillin-streptomycin (Gibco, Cat# 15140122), basic fibroblast growth factor (20 ng/mL, Peprotech, Rocky Hill, NJ, USA, Cat# 45033), and epidermal growth factor (20 ng/mL, Peprotech, Cat# 31509) in a humidified environment with 5% CO_2_ at 37°C. The day after primary cell extraction, sNSCs tend to aggregate and form neurospheres with regular morphology, high refractive indexes, and no protrusions. We used immunofluorescence to detect expression levels of nestin and paired box gene 6, two characteristic NSC markers (Zhu et al., 2021), which allowed precise identification of NSCs (**Additional Figure 1**). We exchanged half of the proliferation medium with fresh medium every 2 days. sNSCs were passed every 4 days at a ratio of 1:2.

### Cell counting kit-8 test

Cell viability was tested with cell counting kit-8 (CCK-8, APExBIO, Houston, TX, USA, Cat# K1018). sNSCs were plated in 96-well plates at a density of 2 × 10^3^ cells per well and cultured in spontaneous differentiation medium (Dulbecco’s modified Eagle medium/nutrient mixture F-12 with 1% B27, 2% N2, 1% penicillin-streptomycin) overnight and AA (PeproTech, Cat# 120-14E) was added to the medium at varying concentrations (5, 10, 15, 25, 50, 75, and 100 ng/mL) for 24 hours. The medium was treated as a blank, and untreated cells served as the control group. Cells were incubated with 10 μL CCK-8 solution for another 4 hours at 37°C. Absorbance was measured at a wavelength of 450 nm via a microplate reader (Thermo Fisher Scientific, Waltham, MA, USA).

### Terminal deoxynucleotidyl transferase-mediated dUTP nick end labeling staining

sNSCs (2.5 × 10^5^ cells/mL) were plated on 12-well plates that were pretreated with 60 μg/mL poly-L-ornithine (PLO, Sigma-Aldrich, St. Louis, MO, USA, Cat# P3655) overnight under spontaneous differentiation conditions and stimulated with 100 mM H_2_O_2_ for 4 hours. The corresponding volume of phosphate-buffered saline was added to the control group. The original medium was removed and the wells were washed twice with phosphate-buffered saline (PBS). Spontaneous differentiation medium containing varying concentrations of AA (10, 15, and 25 ng/mL) was added and incubated for 24 hours. Apoptosis was detected in each group using the terminal deoxynucleotidyl transferase-mediated dUTP nick end labeling (TUNEL) assay kit (Beyotime, Shanghai, China, Cat# C1091). Bright-field images and TUNEL staining images of cells before and after AA treatment were obtained by an inverted fluorescence microscope (Nikon, Tokyo, Japan).

### Apoptosis analysis

Oxidative stress was induced in sNSCs-induced neuron via stimulation with 100 mM H_2_O_2_. Subsequently, the sNSCs-induced neurons were treated with AA (10, 15, and 25 ng/mL) for 24 hours. Accutase (STEMCELL, Vancouver, BC, Canada, Cat# 07920) was added to collect single cells, and the Annexin V-fluorescein isothiocyanate (FITC)/propidine iodide (PI) double-staining apoptosis detection kit (KeyGEN BioTECH, Nanjing, China, Cat# KGA1102-100) was used for apoptosis analysis. The cells were filtered with a 200-mesh and analyzed by flow cytometry (Amnis® ImageStream®XMk II, Luminex, Seattle, WA, USA).

### Animals

To maintain the consistency of the model (Marinelli et al., 2019) while reducing the loss of animals due to urinary tract infections, we used 8-week-old female C57BL/6 mice (**~**20 g) in this study. All mice were housed under at 25 ± 1°C and 50% ± 5% humidity on a 12-hour light/dark cycle. Food and water were provided *ad libitum*. Surgeries were performed after a 1-week adaptation period.

### Spinal cord injury modeling

We used a contusion SCI model in all experiments. The surgical procedure was performed according to a previous report (Zhao et al., 2022). Briefly, mice were immobilized on a rodent standard brain stereotaxic instrument (RWD) and anesthetized with 2% to 3% isoflurane. The thoracic vertebrae (T9–T11) were exposed, followed by a T10 laminectomy. With the corresponding region of T10 as the injury area and the posterior median vessels as the center, an acute spinal cord contusion injury model was prepared using a percussion device (W.M. Keck, New Haven, CT, USA) with a 5 g × 12.5 mm strength. For the sham group, 10 randomly selected mice underwent a similar surgical procedure in which the vertebral plate was removed, but without any subsequent contusion. For all mice, saline was used for repeated rinsing to check for active bleeding. After complete hemostasis, the muscle and skin were sutured. The mice were placed on a thermostatic plate, kept warm for 2 hours, and then returned to the cage after complete awakening; artificial urination was performed twice daily until the bladder function normalized. The non-sham animals were then randomized and divided into two groups: the SCI-only group (*n* = 10), which comprised SCI mice with no further treatments and the SCI-AA group (*n* = 10), which comprised SCI mice that would be treated with intrathecal AA injection.

### Drug administration

AA group animals were given intrathecal AA (Peprotech, Cat# 120-14E) injections at 2 hours, 2 days, 5 days, and 7 days after SCI surgery. The procedure was as follows: mice were anesthetized with isoflurane (2%) in oxygen-enriched air and then fixed on a stereotaxic instrument. A 1.5 cm incision was made along the midline of the mouse lumbar skin. A sterile needle (30 G/0.3 × 13 mm) was fixed on a micro-syringe (Hamilton, Reno, NV, USA) containing AA solution. The needle was inserted into the lumbar vertebra at the midline of the spine at an angle of 70°. When the needle touched the bone, the angle of the needle was adjusted to a 30° pinch angle, and the needle was inserted between the vertebral segments. The needle was correctly inserted when there was no resistance to the needle being pushed into the spine, and a reflexive popping of the tail or hind legs occurred. The plunger of the syringe was gently pushed and 20 µL of AA solution was slowly injected into the subarachnoid space of the spinal cord at a delivery concentration of 1 µg/kg. The injection time was controlled to last 1 minute. After the injection, the needle was kept in place for another minute to allow for adequate absorption.

### Behavioral evaluation

We used Basso mouse scale (BMS) open-field ratings to assess the recovery of locomotor function. Findings on the BMS scoring system has shown that before the SCI surgery, animals scored a 9 on the BMS, with frequent plantar stepping and stable gait condition, while after the surgery, they scored a 0 and had no ankle movement implying the successful establishment of a SCI model (Basso et al., 2006). In the current study, mice were pre-positioned in an open field for 2 minutes to acclimatize and then were evaluated for 3–5 minutes using the double-masked principle. Postoperative BMS scores were performed weekly for 8 weeks. The body weights of each mouse were measured by electronic weighing scales (Zhengfeng, Chongqing, China) and weights were recorded after each BMS assessment.

### Electrophysiological assay

Motor-evoked potentials (MEPs) were analyzed 8 weeks after the operation. Keypoint II dual-channel evoked potential/electromyography (AlphaLab SnR, Alpha Omage, Alpharetta, GA, USA) was used to record MEP signals in each group. The procedure is described below: The mice were anesthetized via intraperitoneal injection of 4% pentobarbital sodium by weight (45 mg/kg, Sigma-Aldrich, Cat# P3761) and placed on the operating table for further preparation. The mice were shaved and sterilized with alcohol, and the motor area of the cerebral cortex was subsequently exposed. The positive electrodes, which served as stimulating electrodes, were positioned on the skull surface of the motor area of the cerebral cortex following previously described methodology (Li et al., 2020). The recording electrode was inserted into the gastrocnemius muscle of the left or right hind limb at a depth of approximately 1.5 mm. Concurrently, the reference electrode was placed 2 cm away from the recording electrode, and the earth wire was placed between the stimulating and recording electrodes. During the experiment, a single square wave pulse (1 Hz) with an intensity of 0–10 mA was employed to stimulate the motor cortex area of the brain, with the resulting motor-evoked potentials being recorded. MATLAB (V.2023, MathWorks, Natick, MA, USA) and GraphPad Prism 9.5 (GraphPad Software, Boston, MA, CA, USA) were used to analyze the MEP latency and the waveform amplitude of each group.

### RNA sequencing

Mice in each group were selected randomly for RNA sequencing (RNA-seq) analysis. Approximately 4 mm of spinal cord tissue centered on the injury site was collected on ice and then immediately transferred to liquid nitrogen. Subsequently, the samples were sent to the Beijing Genomics Institute (BGI) for total RNA isolation and RNA-seq analysis. The results were analyzed and visualized using the BGI online analytics platform (https://eu-biosys.bgi.com/#/report/login). Kyoto Encyclopedia of Genes and Genomes (KEGG), Gene Ontology (GO), and Gene Set Enrichment Analysis (GSEA) were used for gene annotation and further screening of key genes and pathways.

### Immunofluorescence staining

Immunofluorescence staining of tissue samples was performed as follows: mice were anesthetized with an overdose of pentobarbital sodium (45 mg/kg body weight) and perfused with phosphate-buffered saline followed by 4% paraformaldehyde (Solarbio, Beijing, China, Cat# P1110). The spine within the injury site was extracted and then fixed in 4% paraformaldehyde at 4°C overnight. The tissues were sequentially dehydrated in 15% and 30% sucrose solution. The spinal cord tissues were embedded in Tissue-Tek® O.C.T. Compound (Sakura Finetek, Torrance, CA, USA, Cat# 4583) and stored at –80°C. 10-μm-thick frozen sections were cut by a Leica CM3050S cryostat microtome (Leica, Wetzlar, Germany). Tissue slices were permeabilized and blocked, then incubated with primary antibodies at 4°C overnight, followed by 1 hour of fluorescent secondary antibody incubation at room temperature, and 4′,6-diamidino-2-phenylindole dihydrochloride (MedChemExpress, Monmouth Junction, NJ, USA) was used to mark the cell nuclei.

sNSCs were fixed with 4% paraformaldehyde and then permeabilized with 0.3% Triton X-100. After being blocked in 5% donkey serum for an hour, the cell coverslips were incubated with the primary antibody at 4°C overnight and subsequently incubated with fluorescent secondary antibody and 4′,6-diamidino-2-phenylindole dihydrochloride (1 hour at room temperature). All tissue sections and cell coverslips were imaged using a confocal laser scanning microscope (LSM 880, Zeiss, Oberkochen, Germany). All fluorescence images were quantified by calculating the average fluorescent signal intensity in ZEN 2 (blue edition, Zeiss) and the specific analysis steps were as follows: (1) Open the captured image with the Zeiss ZEN software (blue version); (2) Use “Graphics”-“Draw Rectangle” to draw a square on each image (always the same size), providing the mean intensity values of each image; (3) Use ‘Graphics’ - ‘Draw Rectangle’ to draw a square of the same size in each image in an area where no visible signal was present. This provided the average background fluorescent intensity for background removal in subsequent steps. The antibodies used are listed in **Additional Table 1**.

**Additional Table 1:** Information of antibodies

Target	Host species	Dilution	Company	Catalog number	RRID	Application
βIII-TUB	Rabbit	1:200	Cell Signaling Technology, Danvers, MA, USA	5568S	AB_10694505	WB, IF
NeuN	Guinea pig	1:1000	Millipore, Billerica, MA, USA	ABN90P	AB_2341095	IF
Map2	Rabbit	1:1000	Abcam, Cambridge, MA, USA	Ab183830	AB_2895301	IF, FC
GFAP	Chicken	1:500	Abcam	Ab4674	AB_304558	WB, IF
5-HT	Rabbit	1:100	Immuonstar, Hudson, WI, USA	20080	AB_572263	WB, IF
NF200	Rabbit	1:1000	Cell Signaling Technology	30564	AB_2616038	WB, IF
488-conjugated LC3-I/II	Rabbit	1:200	Proteintech, Wuhan, China	CL488-14600	AB_2919097	IF, FC
Sema3A	Rabbit	1:200	Bioss, Beijing, China	Bs-1121R	AB_10855398	WB, IF
Sema3A	Mouse	1:500	ABclonal, Woburn, MA, USA	A12967	AB_2759814	WB, IF
Nrpl	Mouse	1:50	Santa Cruz Biotechnology, Santa Cruz, CA, USA	Sc-5307	AB_2282634	WB, IF
LC3A/B	Rabbit	1:1000	Cell Signaling Technology	12741	AB_2617131	WB, IF
P62	Rabbit	1:10000	Abcam	ab109012	AB_2810880	WB, FC, IF
Beclinl	Rabbit	0.5-2 μg/mL	Sigma-Aldrich, St. Louis, MO, USA	PRS3613	AB_2619135	WB, IF
PI3K	Rabbit	1:1000	Cell Signaling Technology	4249S	AB_2165248	WB, IF
PI3K	Rabbit	1:1000	Abcam	ab302958	AB_2797606	WB, IF
AKT	Rabbit	1:1000	Cell Signaling Technology	4691	AB_915783	WB, IF, FC
p-AKT	Rabbit	1:1000	Cell Signaling Technology	13038	AB_2629447	WB, IF, FC
mTOR	Rabbit	1:1000	Cell Signaling Technology	2983S	AB_2105622	WB, IF, FC
p-mTOR	Rabbit	1:1000	Cell Signaling Technology	5536	AB_10691552	WB, IF
β-Actin	Mouse	1:5000	Proteintech	66009-1	AB_2687938	WB, IF, FC
Anti-Rabbit IgG (H+L) Highly Cross-Adsorbed Secondary Antibody, Alexa Fluor™ Plus 488	Donkey	1:500	Invitrogen, Carlsbad, CA, USA	A32790	AB_2762833	WB, IF
Anti-Rabbit IgG (H+L) Highly Cross-Adsorbed Secondary Antibody, Alexa Fluor™ Plus 555	Donkey	1:500	Invitrogen	A32794	AB_2762834	WB, IF
Anti-Rabbit IgG (H+L) Highly Cross-Adsorbed Secondary Antibody, Alexa Fluor™ Plus 647	Donkey	1:500	Invitrogen	A32795	AB_2762835	WB, IF

5-HT: 5-Hydroxytryptamine; AKT: protein kinase B; FC: flow cytometry; GFAP: glial fibrillary acidic protein; IF: immunofluorescence; LC3: microtubule-associated proteins light chain; Map2: microtubule-associated protein 2; LC3: microtubule-associated proteins light chain; NeuN: neuronal nuclei antigen; NF200: neurofilament-200; Nrp1: neuropilin 1; PI3K catalytic subunit gamma; p-AKT: PI3K catalytic subunit gamma; p-mTOR: phospho-mTOR; Sema: semaphorin; WB: western blot.

### Quantitative polymerase chain reaction

Total RNA was extracted from sNSCs and spinal cord tissues using RNAiso Plus (Takara Biomedical Technology, Beijing, China, Cat# 9108) reagent according to the manufacturer’s protocol. NanoDrop ND-2000 spectrophotometer (Thermo Fisher Scientific) was used to detect RNA quality and concentration. Reverse transcription of RNA was performed using the primer script reverse transcriptase kit (Takara Biomedical Technology, Cat# 2690S), and quantitative polymerase chain reaction (qPCR) was conducted via TB Green Premix Ex Taq (Takara Biomedical Technology, Cat# RR820A) on a QuantStudio 3 Real-Time PCR instrument (Thermo Fisher Scientific). The multiple changes were computed using the 2^–ΔΔCt^ technique after normalizing the transcription level of the housekeeping gene GAPDH (Livak and Schmittgen, 2001). The qPCR primers used are presented in **Additional Table 2**.

**Additional Table 2:** Primer used for quantitative polymerase chain reaction

Gene name	Primer name	Sequence (5' to 3')
*Atg13*	Ms-Atg13-F	GCATTCAT GTCCAC CAGGCAAT
	Ms-Atg13-R	GTGTCCGTCACCACAGGAGTAG
*ULK1*	Ms-ULK1-F	AAGTTCGAGTTCTCTCGCAAG
	Ms-ULK1-R	CGATGTTTTCGTGCTTTAGTTCC
*Pik3cg*	Ms-Pik3cg-F	CACTGGAGTCACCGGCTAC
	Ms-Pik3cg-R	GACACTGTGAACACACTCTCG
*Pik3r5*	Ms-Pik3r5-F	TGCTCTGGAGCGATGCTTG
	Ms-Pik3r5-R	ACCTCTTGGGTCTTTTGTAGGA
*Pik3cd*	Ms-Pik3cd-F	GTAAACGACTTCCGCACTAAGA
	Ms-Pik3cd-R	GCTGACACGCAATAAGCCG
*Egfr*	Ms-Egfr-F	GCCATCTGGGCCAAAGATACC
	Ms-Egfr-R	GTCTTCGCATGAATAGGCCAAT
*SEMA3a*	Ms-SEMA3a-F	GGCTGGTTCACTGGGATTG
	Ms-SEMA3a-R	CCGTTTGCATAGTTTGCTCTGG
*SEMA3b*	Ms-SEMA3b-F	GTAGCAGGGCTAGGGGATACT
	Ms-SEMA3b-R	AAGGCTTCATAACAGCAGGTC
*SEMA3c*	Ms-SEMA3c-F	ATGGCATTCCGGGCGATTT
	Ms-SEMA3c-R	GGTTTTGGTTTCTCGAAGCTCA
*SEMA3d*	Ms-SEMA3d-F	CTGTATCCCCTTTTTGGGTTCAT
	Ms-SEMA3d-R	AACCAGACTGAGCAGGAAGAC
*Nrp1*	Ms-Nrp1-F	GACAAATGTGGCGGGACCATA
	Ms-Nrp1-R	TGGATTAGCCATTCACACTTCTC
*SP1*	Ms-SP1-F	GCCGCCTTTTCTCAGACTC
	Ms-SP1-R	TTGGGTGACTCAATTCTGCTG
*TFEB*	Ms-TFEB-F	CCACCCCAGCCATCAACAC
	Ms-TFEB-R	CAGACAGATACTCCCGAACCTT
*GAPDH*	Ms-GAPDH-F	AGGTCGGTGTGAACGGATTTG
	Ms-GAPDH-R	TGTAGACCATGTAGTTGAGGTCA

All species are mouse (Ms). Atg 13: autophagy-related protein 13; Egfr: epidermal growth factor receptor; F: forward; GAPDH: glyceraldehyde 3-phosphate dehydrogenase; Nrp1: neuropilin 1; Pik3cd: PI3K catalytic subunit delta; Pik3cg: PI3K catalytic subunit gamma; Pik3r5: PI3K regulatory subunit 5; R: reverse; SEMA: semaphorin; SP1: specificity protein 1; TFEB: transcription factor EB; ULK1: UNC-51-like kinase 1.

### Western blot analysis

Proteins were extracted from animal spinal cord tissue and whole sNSCs using whole cell lysis assay kit (KeyGEN BioTECH, Cat# KGB5303-100). All sample concentrations were measured using a bicinchoninic acid assay protein assay kit (KeyGEN BioTECH, Cat# KGP903). Twenty micrograms of protein were loaded and separated by sodium dodecyl sulfate-polyacrylamide gel electrophoresis, then transferred onto polyvinylidene difluoride membranes (Millipore, Billerica, MA, USA, IPVH00010). After a 1-hour block in 5% bovine serum albumin (Solarbio, Cat# A8020), membranes were incubated with specific primary antibodies overnight at 4°C. Subsequently, the membranes were washed three times by Tris buffered saline with Tween-20 and incubated with secondary antibodies for 1 hour at room temperature. The antibodies used are listed in **Additional Table 1**. The bands were visualized with a chemiluminescence kit (Millipore, Cat# P90720) and were detected using the Tanon chemiluminescence detection system (Tanon, Shanghai, China).

### Statistical analysis

Immunofluorescence staining results were exported by Zen (Zeiss, Oberkochen, Germany, v2.3, Blue edition). Western blot results were quantified by ImageJ (v2.14.0). All data were collected using three or more independent biological experiments and are shown as mean ± standard error of the mean (SEM). Statistically analysis and visualization were achieved using GraphPad Prism or MATLAB. The statistical differences between two groups of data were analyzed by Welch’s *t*-test; before analyzing the statistical differences of the data between multiple groups, descriptive statistics and distribution analyses were performed on each group of data. For normally distributed quantitative data, statistical analysis was performed by one- or two-way analysis of variance (ANOVA) with Tukey’s multiple comparisons test or Dunnett’s multiple comparisons test. Statistical significance was set as *P* < 0.05.

## Results

### Activin A enhances cell proliferation and neuronal differentiation of neural stem cells

**Figure 1:**
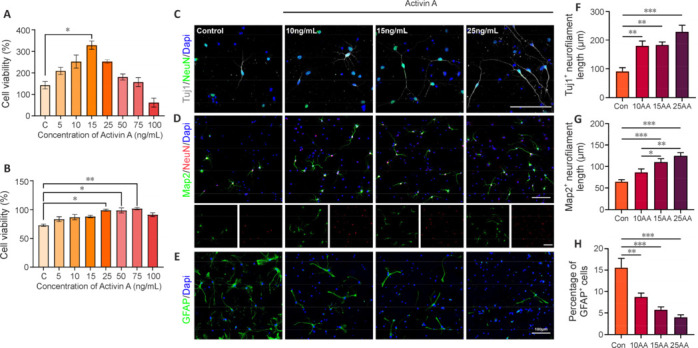
AA promotes the proliferation of NSCs and guides the differentiation of NSCs to neurons. (A) CCK-8 shows the cell viability of sNSCs after 24 hours of AA treatment at different doses under spontaneous differentiation conditions. (B) Same as A, but under proliferation conditions. (C–E) Representative immunofluorescence images of neurons (Tuj1^+^-gray, Alexa Fluor® 555, and Map2^+^-green, Alexa Fluor® 555/NeuN^+^-green or red, Alexa Fluor® 488) and astrocytes (GFAP^+^-green, Alexa Fluor® 488). The number of neurons showed a concentration-dependent significant increase in the AA-treated groups with fewer astrocytes (GFAP^+^ cells). Scale bars: 100 µm. (F, G) Quantitative analysis of neurofilament length of Tuj1^+^ and Map2^+^ cell projections. (H) Quantitative analysis of GFAP^+^ cell number relative to the DAPI^+^. Data are expressed as mean ± SEM (*n* = 3). *P < 0.05, **P < 0.01, ***P < 0.001 (one-way analysis of variance with Dunnett’s multiple comparisons test for A and B; two-way analysis of variance with Dunnett’s multiple comparisons test for F, H, and I). AA: Activin A; CCK-8: Cell Counting Kit-8; Dapi: 4′,6-diamidino-2-phenylindole dihydrochloride; GFAP: glial fibrillary acidic protein; Map2: microtubule-associated protein 2; NeuN: neuronal nuclei antigen; NSCs: neural stem cells; sNSCs: spinal-derived NSCs; Tuj1: neuronal class III β-tubulin.

We investigated how well a range of AA concentrations affected the proliferative capacity of NSCs under spontaneous differentiation conditions (**Figure 1A**). After 24-hour incubation in AA, sNSCs showed enhanced cell proliferation compared with the control group, with the highest proliferation being in the 15 ng/mL AA group (**Figure 1A**). Interestingly, proliferation decreased at higher AA concentrations, which can be explained by its activation of caspase in a dose-dependent manner, which leads to apoptosis (Chen et al., 2002). Furthermore, we also evaluated sNSC proliferative capacity under proliferation conditions (**Figure 1B**). To investigate the effect of AA on NSC differentiation under differentiation factors-free condition, sNSCs were treated with AA (10, 15, and 25 ng/mL) in a spontaneous differentiation medium for 7 days. Immunofluorescence staining revealed that the quantity of neuronal class III β-tubulin (Tuj1)-positive cells and microtubule-associated protein 2-positive (Map2^+^)/neuronal nuclei antigen-positive (NeuN^+^) cells were both significantly higher in the AA groups than in the control group (**Figure 1C** and **D**). Notably, axons were longer and more numerous in the 25 ng/mL AA group than the other groups, while among themselves, the other groups showed no significant difference in axon extension (**Figure 1C** and **D**). Concurrently, there was a marked reduction in the population of glial fibrillary acidic protein (GFAP)-positive cells in the AA-treated groups, indicating that AA treatment biased NSC differentiation towards neurons rather than glial cells (**Figure 1E–H**). These findings underscore the potential that specific concentrations of AA have to stimulate the proliferation and neuronal differentiation of NSCs.

### Activin A reduces the cell apoptosis stimulated by oxidative stress

In secondary injury, oxidative stress is associated with apoptosis and neuroinflammation, and its development can increase complications, consequently worsening the injury after the initial SCI. Therefore, inhibiting oxidative stress and alleviating its symptoms seems to be a potential therapy for SCI (Zhang et al., 2023a).

**Figure 2:**
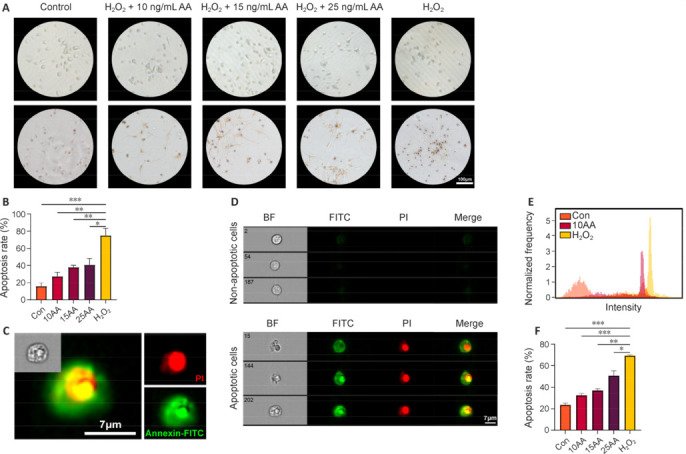
AA alleviates the apoptosis-mediated by oxidative stress *in vitro*. (A) Representative bright-field images (upper row) and TUNEL stained images (bottom row) for each group after 24-hour AA treatment following induction of oxidative stress. H_2_O_2_ stimulation caused neuronal cytosol crumpling and neurofilament reduction, which was attenuated by the administration of AA. (B) The quantification of the TUNEL-positive cells in each group (*n* = 3). (C) Representative Annexin V-FITC^+^/PI^+^ merge image of apoptotic cells. (D) Representative images of non-apoptotic cells (top row) and apoptotic cells (bottom row) were captured using an imaging flow cytometry system. High-intensity FITC and PI fluorescent signals were detected in the cytosol after treatment with H_2_O_2_. Scale bars: 7 µm in C and D. (E) Histogram showing the relative fluorescence intensity of PI in the control, 10 ng/mL AA treatment, and H_2_O_2_ groups. (F) Apoptosis rates in each group as detected by flow cytometry with Annexin V-FITC/PI double staining. Data are expressed as mean ± SEM (*n* = 3). *P < 0.05, **P < 0.01, ***P < 0.001 (one-way analysis of variance with Tukey’s multiple comparisons test for B and F). AA: Activin A; BF: bright field; FITC: fluorescein isothiocyanate; PI: propidium iodide; TUNEL: dT-mediated dUTP nick-end labeling.

Neurons differentiated from sNSCs were stimulated with H_2_O_2_ to establish an *in vitro* oxidative stress model. The cell morphologies of neurons in each group after stimulation with 100 mM H_2_O_2_ for 4 hours and drug treatment for 24 hours are shown in **Figure 2A**. Compared with those in the control group, neurons stimulated with H_2_O_2_ exhibited shortened neurofilaments, wrinkled cytosol, or hyaline globules, indicating that H_2_O_2_ successfully induced apoptosis in neurons. All AA-treated groups showed greater neuronal structural integrity than the H_2_O_2_-only group. To further quantify AA’s ability to reduce apoptosis, we used TUNEL and flow cytometry to assess the percentage of cell apoptosis for each group (**Figure 2B–F**). Compared with the control group, the H_2_O_2_-only group showed a dramatically distinct FITC/PI double-positive signal (**Figure 2D**). Both methods of analysis revealed a smaller proportion of apoptotic cells in the AA-treated groups than in the H_2_O_2_-only group, and the percentage of apoptotic cells differed significantly depending on the dose of AA (**Figure 2B** and **F**).

### Activin A promotes motor function recovery and neural circuit reconstruction

**Figure 3:**
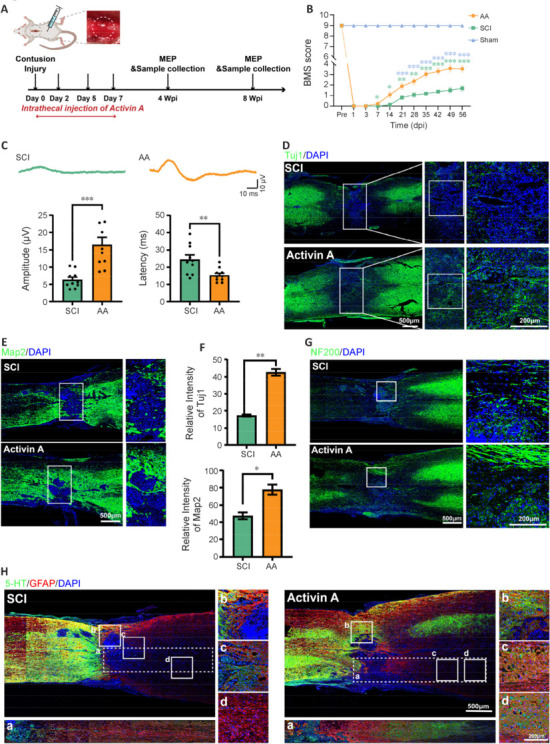
AA promotes locomotor recovery and neural circuit integrity in the mouse model of SCI. (A) Schematic diagram of the experimental design for AA therapy and electrophysiological assays in the SCI contusion model. (B) BMS scores from 1 day to 8 weeks after surgery in each group. (C) Analysis of the electrophysiological data (MEP amplitude and latency) for the AA-treatment and SCI groups performed 8 weeks postoperatively (*n* = 10). (D, E) Immunofluorescence images of Tuj1-positive cells (D, green, Alexa Fluor® 488) and Map2-positive cells (E, green, Alexa Fluor® 488) in longitudinal spinal cord sections 8 weeks after surgery. A significantly larger positive signal for neuronal markers was observed in the injury center of the AA-treated group compared with the findings in the SCI group. (F) Quantification of the fluorescence intensity in D and E. Data are expressed as mean ± SEM (*n* = 3). *P < 0.05; **P < 0.01; ***P < 0.001 (two-way analysis of variance with Dunnett’s multiple comparisons test for B; two-tailed unpaired *t*-test for C and F). (G) Representative images of neurofilament marker (NF200, green, Alexa Fluor® 488) expression in longitudinal spinal cord sections. Compared with the SCI group, the AA-treated group exhibited more neurofilaments across the injury center. (H) Immunofluorescence staining of GFAP (red, Alexa Fluor® 647) and 5-HT (green, Alexa Fluor® 488). A significant 5-HT signal was observed downstream of the lesion region in the AA-treated group. Scale bars: as shown in the Figure. 5-HT: 5-Hydroxytryptamine; AA: Activin A; DAPI: 4’,6-diamidino-2-phenylindole dihydrochloride; Dpi: day(s) post-injury; GFAP: glial fibrillary acidic protein; MEP: motor-evoked potential; NF200: neurofilament-200; SCI: spinal cord injury; Tuj1: neuronal class III β-tubulin; Wpi: week(s) post-injury.

According to the findings reported above, AA treatment promoted sNSCs proliferation and neural differentiation and decreased apoptosis caused by oxidative stress *in vitro*. To investigate AA function *in vivo*, mouse models of contusion SCI were treated with AA (**Figure 3A**). A previous study indicated that peak cell death and autophagy occurs in the acute phase (within 24 hours post-injury) (Kanno et al., 2009), and that administering AA at the onset of increased autophagic activity may assist in the restoration of autophagic homeostasis, thereby attenuating cell death. Therefore, the first AA treatment was administrated 2 hours post-injury to counteract immediate autophagic responses; continuous re-injection at 2, 5, and 7 days post-injury aimed to facilitate functional recovery and prevent late-onset cell death associated with chronic autophagy dysregulation. Notably, there was no significant weight loss in mice at 8 weeks after AA treatment, demonstrating that the treatment was safe (**Additional Figure 2**). The BMS was used to evaluate motor function recovery after SCI (**Figure 3B** and **Additional Videos 1** and **2**). After four AA treatments, motor function in the SCI mice was significantly improved, as the BMS score showed. Furthermore, an electrophysiological assay was performed to evaluate neural circuit integrity by stimulating hind-limb motor cortex and recording hind limb MEPs. As shown in **Figure 3C**, the mean MEP amplitudes 8 weeks after injury were was 2.6-times higher in the SCI-AA group (16.48 ± 2.142 μV) than in the SCI-only group (6.322 ± 0.775 μV), while MEP latencies were 1.6-times lower (AA-15.36 ± 1.228 ms; SCI-24.39 ± 2.687 ms). The immunofluorescence analysis at the lesion site revealed more neuron-related proteins (Tuj1, Map2, and neurofilament protein NF200) in the SCI-AA group, confirming AA’s ability to protect neurons and promote their reconstruction (**Figure 3D–G**). Furthermore, immunofluorescence analysis of axonal markers (5-hydroxytryptamine) revealed a significant signal in the downstream region of the damaged site in the AA-treated tissue samples (**Figure 3H**). These findings suggest that AA treatment can restore damaged neural circuits in a mouse model of SCI, thereby rescuing the reduced locomotor function.

### Activin A activates the PI3K pathway

**Figure 4:**
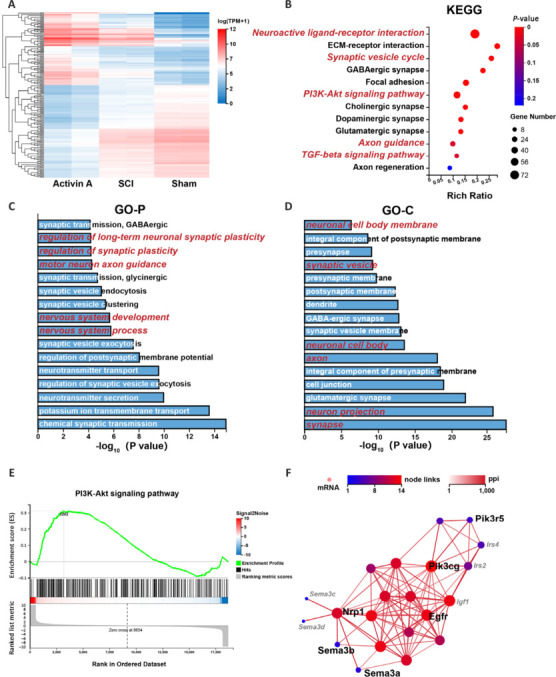
RNA sequencing analysis of AA-treated mice after SCI. (A) Heat map showing the mRNA expression of DEGs for the AA and SCI groups. (B) KEGG pathway analysis of DEGs showing enrichment of activated pathways after AA treatment. (C, D) GO-P and GO-C enrichment of DEGs. (E) GSEA analysis of the PI3K/AKT pathway. (F) PPI analysis of key DEGs involved in the PI3K/AKT pathway during AA treatment *in vivo*. AA: Activin A; AKT: protein kinase B; DEGs: differentially expressed genes; GO-C: Gene Ontology cell composition; GO-P: Gene Ontology biological process; GSEA: Gene Set Enrichment Analysis; KEGG: Kyoto Encyclopedia of Genes and Genomes; PI3K: phosphoinositide-3-kinase; PPI: protein-protein interaction network; SCI: spinal cord injury.

To identify the mechanism underlying the observed motor function recovery afforded by AA therapy, we performed RNA-seq to analyze the essential genes and the involved activated pathways. The heatmap depicted in **Figure 4** shows the significant differentially expressed genes (DEGs) between the SCI-AA and SCI-only groups. KEGG pathway analysis based on DEGs showed that the DEGs were primarily enriched in neurotransmission and synaptic transmission pathways, such as neuroactive ligand-receptor interaction and synaptic vesicle cycle pathways (**Figure 4B**). GO enrichment analysis also revealed DEG enrichment in synaptic transmission- and axonogenesis-related pathways (**Figure 4C** and **D**). Meanwhile, activation of the PI3K/AKT signaling pathway differed significantly between SCI-AA and SCI-only groups. GSEA analysis indicated that activation of the PI3K/AKT signaling pathway might be the essential mechanism for AA treatment to exert its neuroprotective effect (**Figure 4E**). Meanwhile, the protein-protein interaction network analysis pointed to vital genes corresponding to the PI3K/AKT signaling pathway (**Figure 4F**).

**Figure 5:**
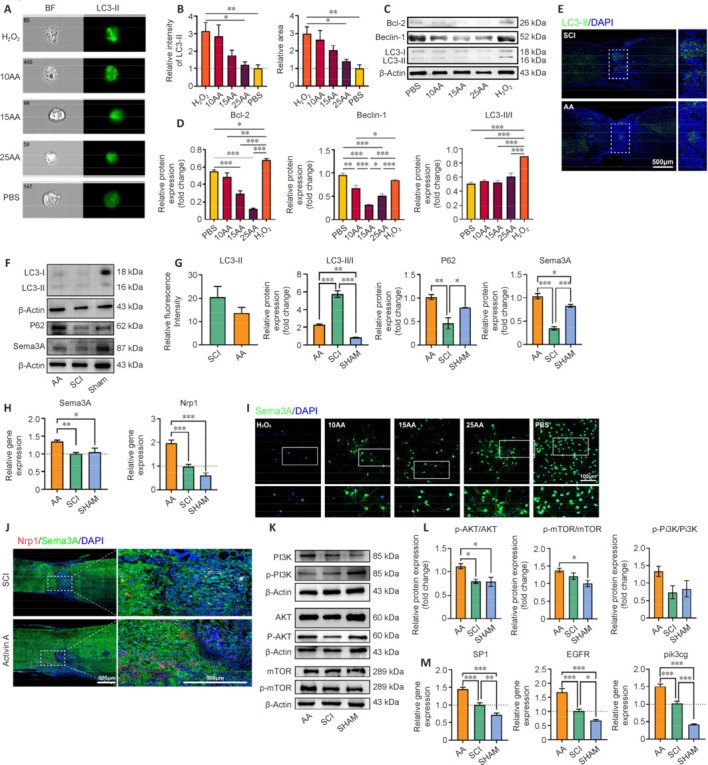
AA alleviates excessive autophagy via activation of the PI3K pathway. (A, B) Intracellular deposition of the autophagy marker LC3-II captured by imaging flow cytometry. Increased LC3-II deposition was observed in the cytosol of H_2_O_2_-treated cells, which was alleviated by AA treatment. Bar plots show each group’s relative fluorescence intensity and LC3-II deposition area (*n* = 6). (C) Western blot images show the expression level of autophagy-related proteins in cell samples. (D) Quantitative analysis of the expression content of autophagy-related proteins in C. (E) Representative immunofluorescence images of LC3-II (green, Alexa Fluor® 488) deposition in spinal cord sections. The injury center in the AA treatment group had a smaller LC3-II deposition area than did controls. (F) Representative western blot images of autophagy markers (LC3 I/II, and P62) and a semaphorin family member (Sema3A) in spinal cord tissue. (G) Quantitative analysis of fluorescence intensity in E and WB bands in F. (H) qPCR showing the expression level of the Sema3A and receptor protein Nrp1 (*n* = 3). (I) Representative immunofluorescence images of Sema3A^+^ (green, Alexa Fluor® 488) cells after H_2_O_2_ stimulation and AA treatment. Sema3A was highly expressed in neuronal cytosol and axons. Cells that received AA treatment had higher Sema3A expression compared with those in the H_2_O_2_ group, and the increase was concentration-dependent. (J) Representative immunofluorescence images of Nrp1 (red, Alexa Fluor® 555) and Sema3A (green, Alexa Fluor® 488) in spinal cord sections. The AA-treated group exhibited more pronounced Sema3A/Nrp1-positive signals at the injury site and a more intact tissue junction than did the SCI group. (K) Western blot images showing the expression levels of PI3K/AKT/mTOR pathway-related proteins. (L) Quantitative analysis of the expression levels of PI3K/AKT pathway-related proteins in K. (M) The expression levels of EGFR/PI3K pathway-related genes by qPCR (*n* = 3). Data are expressed as mean ± SEM. *P < 0.05, **P < 0.01, ***P < 0.001 (one-way analysis of variance with Tukey’s multiple comparisons test for B, D, and L; one-way analysis of variance with Dunnett’s multiple comparisons test for H and M). Scale bars: as shown in the Figure. AA: Activin A; AKT: protein kinase B; BF: bright field; DAPI: 4′,6-diamidino-2-phenylindole dihydrochloride; EGFR: epidermal growth factor receptor; LC3: microtubule-associated proteins light chain; mTOR: mammalian target of rapamycin; Nrp1: neuropilin 1; p-AKT: phospho-AKT; p-mTOR: phospho-mTOR; p-PI3K: phospho-PI3K; PBS: phosphate-buffered saline; PI3K: phosphoinositide-3-kinase; pik3cg: PI3K catalytic subunit gamma; qPCR: quantitative polymerase chain reaction; SCI: spinal cord injury; Sema: semaphorin; SP1: specificity protein 1; WB: western blot.

### Activin A exerts neuroprotective effects by inhibiting autophagy hyperactivation via the PI3K/AKT pathway

To investigate the potential impact of AA treatment on autophagy dysregulation following SCI, we used imaging flow cytometry combined with fluorescently labeled LC3-II protein antibody to detect autophagosome deposition level in H_2_O_2_-AA treated cells. LC3-I typically manifests as a diffused signal in the cell, predominantly distributed within the cytoplasm. This form represents the free state of LC3, unbound to the membrane. Conversely, LC3-II exhibits punctate staining, typically clustered on the autophagosome membrane. This punctate signal signifies the formation of autophagosomes and can reflect augmented autophagic activity. As shown in **Figure 5A** and **B**, the relative fluorescence intensity and the deposition area of LC3-II were significantly decreased by different concentrations of AA (10, 15, and 25 ng/mL) compared with those in the H_2_O_2_-only group. Detection of autophagy-related protein expression levels *in vivo* and *in vitro* also indicated that AA therapy can significantly down-regulate autophagy following severe tissue/cell damage, as reflected by the accumulation of P62 in the AA-treated group accompanied by a reduction in LC3-II deposition (**Figure 5C–G**).

According to the RNA-seq analysis, we observed upregulation of semaphorins (Semas) family members in SCI-AA group (**Figure 4F**). Semaphorins are a group of secreted or membrane-associated signaling proteins that participate in physiological processes such as neural development, organ formation, immune response, and angiogenesis, of which types III and IV are widely observed in the human body (Behar et al., 1996). As previously reported, semaphorins III (Sema3) is mainly expressed in neuronal systems and functions in axon guidance and synaptic plasticity. Sema3A has been reported to suppress autophagy when treating cardiac hypertrophy by activating the AKT/mammalian target of rapamycin (mTOR) signaling pathway (Koropouli and Kolodkin, 2014). Our qPCR results showed that mRNA expression of Sema3A and its receptor neuropilin 1 (Nrp1) was significantly higher in the AA group than in the other groups (**Figure 5H**). Therefore, we investigated the expression level of Sema3A in H_2_O_2_-treated neurons (**Figure 5I**). Immunofluorescence analysis showed that Sema3A was expressed in neuronal somata and axons. Compared with the H_2_O_2_-only group, the control group exhibited more Sema3A-positive cells, which appeared to be aggregated and in mutual communication. In contrast, AA-treated groups exhibited a dose-dependent increase in the number of Sema3A-positive cells, indicating a neuroprotective effect of AA. At the same time, the expression of Sema3A and Nrp1 protein, as well as mRNA levels, was upregulated after AA treatment (**Figure 5J** and **Additional Figure 3A**).

A previous study has shown that AA regulates epidermal growth factor receptor expression by activating PI3K/specificity protein 1 through a non-classical pathway, which in turn engages the PI3K/AKT/mTOR pathway to modulate biological processes such as autophagy, cell proliferation, and cell migration (Tsai et al., 2019). This, combined with the previous RNA-seq analysis results, suggests that activating the PI3K/AKT pathway may be essential for AA regulation of autophagy. Western blot and qPCR analyses were conducted to detect the expression levels of critical components in the PI3K/AKT/mTOR pathway at the injury sites (**Figure 5K–M** and **Additional Figure 3B**). The AA group exhibited significant phospho-PI3K, phospho-AKT, and phospho-mTOR upregulation compared with the SCI group (**Figure 5K–L**). Meanwhile, we also detected the expression level of transcription factor EB, a major transcription factor of the autophagy-lysosome pathway, whose activity can be regulated through AKT-mediated phosphorylation (**Additional Figure 4**). However, we found no statistically significant differences in transcription factor EB expression between groups. Because of the complexity of the relationship between AKT and transcription factor EB, a more comprehensive investigation of this issue is warranted in the future.

Overall, these results suggest that AA-mediated activation of the epidermal growth factor receptor/PI3K/AKT pathway is crucial in regulating autophagy levels in SCI mice. In contrast, Sema3A/Nrp1 signaling, in conjunction with the PI3K/AKT pathway, facilitates cell migration and synapse formation and promotes neural circuit reconstruction and motor function recovery in AA-treated SCI in mice.

## Discussion

The pathophysiology of SCI is intricate, with an exaggerated autophagic response hindering the recovery process. This research delves into the therapeutic potential of AA—a growth factor intricately linked to embryonic development and tissue growth—in suppressing autophagy and facilitating the restoration of impaired neurological function following SCI.

The pathophysiological progression of SCI has primary and secondary injury phases. The primary injury, attributed to mechanical trauma, induces axonal and vascular compression, leading to disruption of the blood-brain barrier (Anjum et al., 2020). Subsequently, a sustained secondary injury cascade ensues, involving neuronal and glial cell death, demyelination, oxidative stress, and inflammatory reactions (Iyer et al., 2017; He et al., 2022). To address the intricate pathological microenvironment following SCI and prevent further deterioration at the injury site, developing clinical therapeutic approaches for SCI with composite treatment functions has been a primary research priority.

Neuronal death damages neural circuits. Transplanting exogenous NSCs that can self-renew, migrate, and differentiate into varying types of neurons to the damaged site can replace dead neurons, allowing new synaptic connections to be established and the recovery of neural function (Cao et al., 2022; Guo et al., 2022; Hao et al., 2024). Extensive investigation into spinal cord NSC subgroups reveals that the recruitment and activation of endogenous NSCs into injury areas is emerging as a more productive strategy for spinal cord neural repair (Gilbert et al., 2022; Hosseini et al., 2024). As previously reported, long-term co-cultures with AA promoted the proliferation and differentiation of neural progenitor cells (Rodríguez-Martínez et al., 2012). A study found that AA promotes the specification of ASCL1^+^ rhombomere 1 progenitors to PHOX2A/2B^+^ norepinephrine neuron (NE) precursors in a dose-dependent manner, indicating a specific role of AA in the differentiation of NEs (Tao et al., 2024). In this study, we first validated the pro-proliferative impact of AA on sNSCs. The CCK-8 results show that AA has a proliferative effect on sNSCs, which is concentration-dependent. Additionally, the differentiation assays revealed the potential of AA to assist in the differentiation of NSCs to neurons. We demonstrated that AA promotes NSC differentiation into Map2-positive and Tuj1-positive cells (neurons) instead of glial cells (GFAP^+^ cells). The subsequent RNA-seq results demonstrated that AA activates the PI3K/AKT signaling pathway. Previous findings indicated that the context of PI3K/AKT activation is a significant determinant of cell fate: in neurons, activation typically supports differentiation and survival, whereas in glial cells, activation may promote proliferation without leading to differentiation (Sun et al., 2013). These findings are consistent with the results observed in the present study.

Oxidative stress, another integral part of secondary injury, is caused by transient hypoxia resulting from spinal cord bleeding and is characterized by the generation and accumulation of excessive reactive oxygen species (Duan et al., 2016; Su et al., 2019). The production of many free radicals leads to cytotoxic effects, causing significant neuronal cell death and hindering the repair of neurological function following SCI (Liu et al., 2021). Our investigations on apoptosis using the oxidative stress model indicate that AA protects neurons by reducing oxidative stress-induced apoptosis.

**Figure 6:**
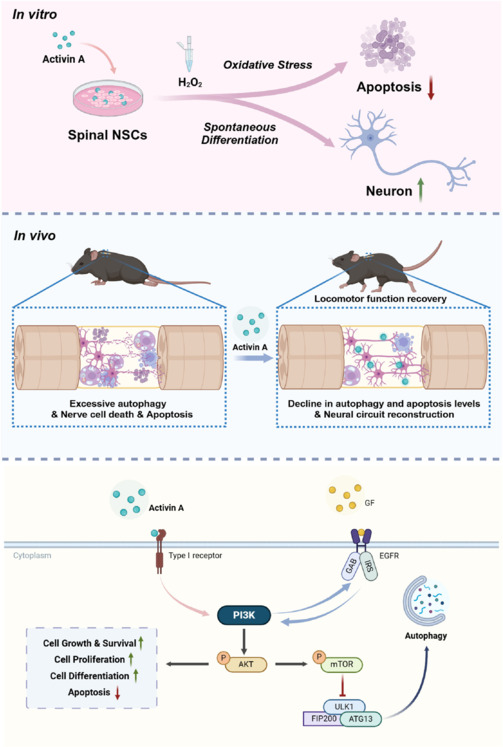
The mechanism whereby activin A mitigates autophagy in the early stages of SCI by activating the PI3K/AKT pathway. Created with BioRender.com. AKT: Protein kinase B; ATG13: autophagy-related protein 13; EGFR: epidermal growth factor receptor; FIP200: FAK family kinase-interacting protein of 200 kDa; GAB: Grb2-associated binder; GF: growth factor; IRS: insulin receptor substrate; mTOR: mammalian target of rapamycin; NSC: neural stem cell; PI3K: phosphoinositide-3-kinase; SCI: spinal cord injury.

Autophagy is a crucial activity that helps regulate normal cellular physiology by eliminating dysfunctional organelles and proteins, thus maintaining cellular homeostasis (Mizushima, 2007). However, autophagy plays a double-edged role in SCI repair: during the early stages of SCI, heightened levels of LC3-II and P62 are observable in the injured tissue, indicating extensive accumulation of autophagosomes, which can exacerbate secondary damage, ultimately leading to neuronal cell death (Gu et al., 2020; Zhang et al., 2020). The abnormal increase of Beclin1 in injured neural tissue was believed to be closely associated with autophagic cell death in SCI (Kanno et al., 2009). In a severe SCI model, administering the autophagy inhibitor Valproic acid effectively reduced autophagy-related cell death and promoted the recovery of neurological function (Hao et al., 2013). Conversely, in the later stages of SCI, the autophagic flux gradually stabilizes and the neuroprotective function of the autophagic pathway within the CNS is reinstated (Li et al., 2019a). The dual functionality of autophagy in SCI repair suggests that precise modulation of autophagy at various stages of SCI progression could be a promising therapeutic strategy. During the initial phase of injury, suppressing the overactivation of autophagic response, thus restoring the autophagic flux, might mitigate inflammation and decrease cell death. In the later stages of injury, stimulating autophagy appropriately to eliminate damaged organelles and protein aggregates could enhance tissue regeneration. In the current study, after administering AA treatments 4 times in the acute phase of the contusion model, we observed neuronal survival in the damaged tissue, reconstruction of neural circuits, and the recovery of motor function in SCI mice (**Figure 6**). Additionally, a significant downregulation of autophagy (reduced LC3-II) was detected in the injured tissues. *In vitro*, an increase in cellular autophagy levels was observed after oxidative stress, which was rescued by application of AA, as indicated by the significantly reduced levels of intracellular LC3-II.

The PI3K/AKT pathway is a crucial regulator of intracellular signaling and plays a vital role in regulating physiological processes such as cell survival, proliferation, and autophagy. As one of the classical pathways regulating autophagy, activation of the PI3K/AKT pathway leads to downstream mTOR pathway activation, which inhibits autophagy (Lipinski et al., 2015; Bisicchia et al., 2017). 3-Methyladenine blocks autophagosome formation and promotes the survival of distal rubrospinal neurons by regulating PI3K expression (Bisicchia et al., 2017). In the current study, expression levels of PI3K/AKT pathway components were significantly greater in the SCI-AA group than in the SCI-only group, and was accompanied by a decrease in autophagy levels.

Recuring damage to neural circuits following SCI prevents recovery of neural function. The semaphorin family of proteins is involved in axon guidance, neural circuit assembly, and dendritic development, which are crucial for the nervous system’s development, maintenance, and plasticity (Koropouli and Kolodkin, 2014). Various semaphorins regulate the excitation/inhibition balance of neural circuits by affecting pre- and post-synaptic membranes and the stability of synaptic connections. Sema3A and Sema3F can modulate the number of excitatory synapses in the cerebral cortex, while Sema4D and Sema4B participate in the assembly of inhibitory synapses (Sahay et al., 2005; Li et al., 2019b). Following tissue injury, the expression patterns of semaphorin family members change: Sema3A is mainly expressed in motor neurons of the spinal cord. After spinal cord transection, the expression level of Sema3A mRNA rapidly decreases, reaching the lowest point approximately 1 day post-transection, primarily because of neuronal cell death; by 28 days post-transection, Sema3A expression level gradually recovers to the average level as a result of upregulation in surviving neurons (Hashimoto et al., 2004). In the current study, we detected significant upregulation of Sema3A and its receptor Nrp1 in spinal cord tissue 8 weeks after injury after treatment with AA, which suggests that AA facilitates the formation of new neural circuits by enhancing neuron survival or promoting the differentiation of endogenous NSCs into neurons after SCI.

Despite the evidence from numerous studies indicating the neuroprotective potential of AA for treating CNS injuries and disorders, some evidence suggests that AA therapy presents certain risks that warrant consideration: AA’s activity is modulated by various factors, including inhibitors like bone morphogenetic proteins. These interactions may limit its effectiveness or lead to unpredictable responses in different biological contexts (Su et al., 2018). Concurrently, in a first-in-human clinical trial of the AA inhibitor STM 434, it was found that overexpression of AA is associated with shortened survival time in various cancers, including ovarian, colon, and gastric cancers, while the application of STM 434 slow down the process. This indicating that in addition to NSCs, AA can also facilitate tumor proliferation and invasion (Tao et al., 2019). Therefore, further research is needed to fully understand these risks and optimize AA’s usage in therapeutic settings.

For subsequent studies, we will further evaluate the anti-inflammatory effects of AA in SCI repair. The KEGG pathway enrichment analysis results indicate that administering AA might activate the TGF-β signaling pathway. Activation of this pathway promotes angiogenesis, inhibits apoptosis after stroke, and reduces inflammation (Zhang et al., 2021a). Furthermore, evidence suggests that activation of the PI3K/AKT/mTOR pathway reduces intracellular reactive oxygen species levels by increasing the expression of antioxidant enzymes, which attenuates cellular damage from oxidative stress (Shiau et al., 2022). Alternatively, it may also promote the synthesis of glutathione peroxidase 4 protein, a key regulator of ferroptosis; its increased expression significantly reduces the accumulation of lipid peroxides, thereby attenuating iron death (Zhang et al., 2021b). Nevertheless, it remains to be investigated whether activating the PI3K/AKT pathway with AA after SCI can further affect apoptosis caused by oxidative stress or iron death.

The current findings provide evidence of AA’s neuroprotective effect against the excessive autophagic response after SCI. However, there are still some risks and limitations of applying exogenous AA in the treatment of SCI. Additional experiments are needed to confirm the effects of sustained treatment as well as the potential carcinogenic risk of AA. Furthermore, the underlying mechanism of AA-mediated neural circuit reconstruction via Sema3A/Nrp1 requires further elucidation. A comprehensive investigation of the molecular mechanisms underlying all of AA’s effects during SCI treatment is crucial for its potential clinical applications.

In conclusion, AA treatment enables SCI repair by promoting endogenous NSC proliferation and differentiation, reducing apoptosis caused by oxidative stress, and helping to repair neural circuits. This investigation provides evidence supporting the use of AA treatment for SCI recovery.

## Additional files:

***Additional Figure 1:*** *Representative immunofluorescence image of Nestin*^*+*^ *(green, Alexa Fluor® 488) and Pax6*^*+*^ *(red, Alexa Fluor® 555) neurospheres.*

Additional Figure 1 Representative immunofluorescence image of Nestin^+^ (green, Alexa Fluor® 488) and Pax6+ (red, Alexa Fluor® 555) neurospheres
Scale bar: 100 μm. Pax6: Paired box gene 6.

***Additional Figure 2:*** *AA increases the body weight of SCI mice.*

Additional Figure 2 AA increases body weight of SCI mice
Data are expressed as mean ± SEM (*n* = 10). *P < 0.05; **P < 0.01; ***P < 0.001 (two-way analysis of variance with Dunnett’s multiple comparisons test). AA: Activin A; SCI: spinal cord injury; Wpi: week post-injury.

***Additional Figure 3:*** *AA increases the mRNA expression levels of the key genes in Sema family members (A) and PI3K/AKT pathway (B) in spinal cord tissues of SCI mice.*

Additional Figure 3 AA increases the mRNA expression levels of the key genes in Sema family members (A) and PI3K/AKT pathway (B) in spinal cord tissues of SCI mice.
Data are expressed as mean ± SEM (*n =* 3). *P < 0.05, **P < 0.01, ***P < 0.001 (one-way analysis of variance with Tukey’s multiple comparisons test). AA: Activin A; AKT: protein kinase B; PI3K: phosphoinositide-3-kinase; SCI: spinal cord injury; Sema: semaphorin.

***Additional Figure 4:*** *AA has no effects on mRNA expression level of TFEB, a transcription factor of autophagy and lysosome biogenesis, in spinal cord tissues of SCI mice.*

Additional Figure 4 AA has no effects on mRNA expression level of TFEB, a transcription factor of autophagy and lysosome biogenesis, in spinal cord tissues of SCI mice.
Data are expressed as mean ± SEM (*n* = 6) and were analyzed by one-way analysis of variance with Tukey’s multiple comparisons tests. AA: Activin A; SCI: spinal cord injury; TFEB: transcription factor EB.

***Additional Table 1:*** *Information of antibodies.*

***Additional Table 2:*** *Primer used for quantitative polymerase chain reaction.*

***Additional Video 1:*** *Activin A_8 week BMS video-50M.*



***Additional Video 2:*** *SCI_8 week BMS video-50M.*



## Data Availability

*All data relevant to the study are included in the article or uploaded as Additional files*.
