# Supplementary figures and images for: Activin A enhances neurofunctional recovery following traumatic spinal cord injury by inhibiting autophagy
Source: Neural Regen Res. 2025 Mar 25;21(6):2485–94. doi: 10.4103/NRR.NRR-D-24-01021 (PMC13211839; doi:10.4103/NRR.NRR-D-24-01021)

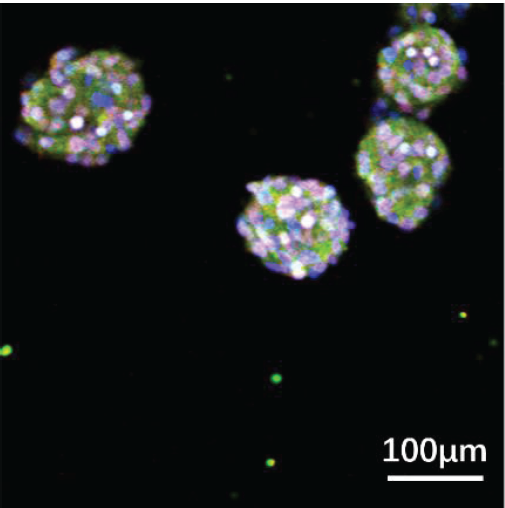

Supplement: Supplementary file 1 [file NRR-21-2485_Suppl1.tif]

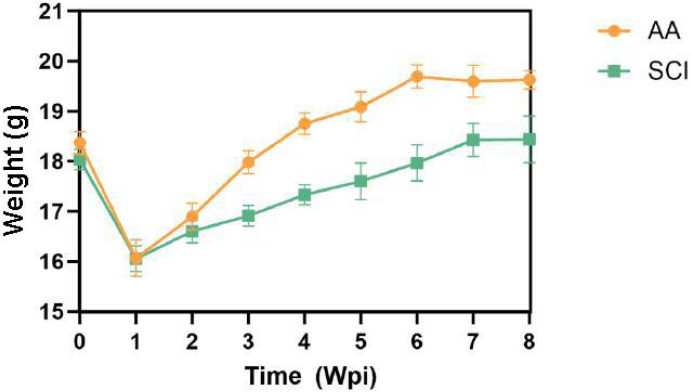

Supplement: Supplementary file 2 [file NRR-21-2485_Suppl2.tif]

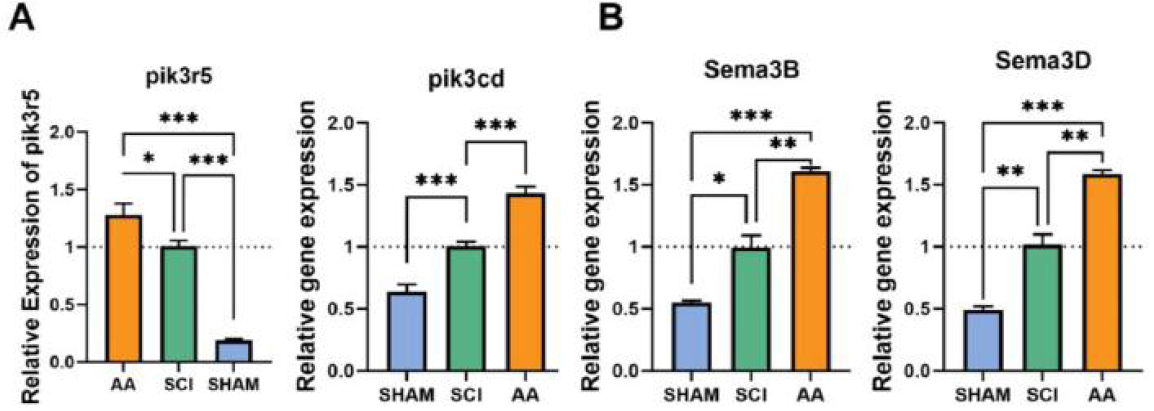

Supplement: Supplementary file 3 [file NRR-21-2485_Suppl3.tif]

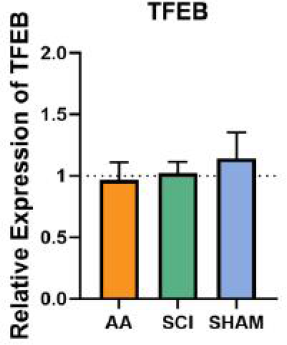

Supplement: Supplementary file 4 [file NRR-21-2485_Suppl4.tif]
